# Rapid direct detection of pathogens for diagnosis of joint infections by MALDI-TOF MS after liquid enrichment in the BacT/Alert blood culture system

**DOI:** 10.1371/journal.pone.0243790

**Published:** 2020-12-11

**Authors:** Christine Noll, Azadda Nasruddin-Yekta, Pia Sternisek, Michael Weig, Uwe Groß, Arndt F. Schilling, Frank Timo Beil, Oliver Bader

**Affiliations:** 1 Institute of Medical Microbiology, University Medical Center Göttingen, Göttingen, Germany; 2 Department for Trauma Surgery, Orthopedics and Plastic Surgery, University Medical Center Göttingen, Göttingen, Germany; 3 Department for Orthopedics, University Medical Center Hamburg-Eppendorf, Hamburg, Germany; Fisheries and Oceans Canada, CANADA

## Abstract

Pathogen identification is a critical step during diagnosis of infectious diseases. Matrix-Assisted Laser Desorption/Ionization Time-Of-Flight mass spectrometry (MALDI-TOF-MS) has become the gold standard for identification of microorganisms cultured on solid media in microbiology laboratories. Direct identification of microbes from liquid specimen, circumventing the need for the additional overnight cultivation step, has been successfully established for blood culture, urine and liquor. Here, we evaluate the ability of MALDI-TOF MS for direct identification of pathogens in synovial fluid after liquid enrichment in BacT/Alert blood culture bottles. Influence of synovial specimen quality on direct species identification with the MALDI BioTyper/Sepsityper was tested with samples inoculated from pretested native synovia with concomitant inoculation of blood or pus, or highly viscous fluid. Here, we achieved >90% concordance with culture on solid medium, and only mixed-species samples posed significant problems. Performance in routine diagnostics was tested prospectively on bottles inoculated by treating physicians on ward. There, we achieved >70% concordance with culture on solid media. The major contributors to test failure were the absence of a measurable mass signal and mixed-specimen samples. The Sepsityper workflow worked well on samples derived from BacT/Alert blood culture bottles inoculated with synovial fluid, giving concordant results to identification from solid media. Host remnant material in the inoculum, such as blood or pus, had no detrimental effect on identification score values of the BioTyper system after processing with the Sepsityper workflow, and neither had the initial viscosity of the synovial sample.

## Introduction

Over the last decades, the numbers of prosthetic joint replacements have continuously increased and are forecast to increase even further with the ageing population [1]. This in turn correlates with a higher number of associated prosthetic joint infections (PJIs). Implant-associated infections have significant consequences on morbidity and mortality and place a high economic burden on the healthcare system [2].

For focused therapy of PIJ, identification of pathogens from synovial fluid is highly relevant. Consequently, aspiration of synovial fluid with microbiological analysis is recommended by the AAOS guide-lines for joint infections [3]. However, several challenges exist in the diagnostic procedures of PJI. Most importantly, samples in association with PJI or bone infections after fracture fixation generally have low bacterial densities with concomitant cellular debris, so that direct bacterial identification out of these samples is difficult [4]. Enrichment steps, e.g. in liquid medium and prolonged incubation (up to 2 weeks), increase sensitivity and specificity [5,6]. They allow observance of slow growing small colony variants [7] or fastidious bacteria, and lead to improved diagnosis [8]. Any possible reduction in processing time will likely have a positive effect on treatment.

For microbes cultured on solid media, Matrix-Assisted Laser Desorption/Ionization Time-Of-Flight Mass Spectrometry (MALDI-TOF MS) has become the gold standard for species identification. This technology has brought significant changes in processing of microbiological probes and replaced most cost- and time intensive phenotypic biochemical assays. For identifying microbial species out of liquid culture, MALDI-TOF is already regularly used for blood specimen (reviewed in [9,10]) and is highly standardized during routine diagnostics through commercialized assays such as the Sepsityper workflow [11]. These procedures circumvent the need for an additional overnight cultivation step. A first study has demonstrated the feasibility of such assays also for orthopedic samples using the BACTEC system [12].

Here, we examined whether BacT/Alert blood culture vials inoculated with synovial fluid were suitable for the Sepsityper workflow, if this yielded microbial identification concordant with the standard culture procedure, and if different levels of host residue influence these results.

## Results and discussion

Several experimental factors influence MALDI-TOF mass spectrum quality when bacteria are analyzed from clinical samples. Residual host material ionizes along with microbial markers. If the host material is in excess, this can lead to spectra representing the host rather than the microbe. Consequently, a main goal of sample pre-processing is eliminating host material, and enriching for microbial cells. A possible option is amplification through liquid culture. This may however introduce several components into the sample interfering with the MALDI-TOF process [13]. In older blood culture systems, charcoal and salt ions from the culture media were shown to interfere with the results [14]. Even after successful amplification, liquid cultures will still contain remnants of host materials, such as blood or tissue cells, soluble proteins, or mucus. These materials generate background noise mass peaks, overlapping with microbial spectra and interfere with the interpretation of bacterial proteome profiles [15]. Therefore, amplicon pretreatment removing host cells and proteins, while further concentrating the microbes, improves spectrum quality.

To test if the residues contained in synovial inoculum had an influence of species identification, we used a total of 23 native synovial samples (Table 1) over a course of 5 weeks, which contained a visible degree of host residue (blood, pus), or were highly viscous. The samples had been analyzed by culture on solid media and were additionally inoculated into pediatric BacT/Alert blood culture bottles. All bottles automatically flagged positive by the next morning (~16h). Using the Sepsityper workflow, 21/23 (91%) of the samples gave concordant results with culture on solid media. Out of these, only two missed the manufacturer-recommended threshold of 2.000 for species-level identifications. Acceptance of lower score values down to 1.700 when using the Sepsityper protocol, however, appear feasible [9]. The two remaining samples (9%) were from mixed-species specimens. In one case (#11) the initial mixed result could not be confirmed by reanalysis, and the Sepsityper identification was concordant with this second culture result (Table 1). Most importantly, there was no apparent hindrance through blood, pus, or high viscosity present in the inoculum.

**Table 1:** Results of spiked culture Sepsityper identification.

**Nr.**	**consistency**	**joint**	**routine identification**	**score**	**Sepsityper identification**
concordant identification to species level (Score ≥ 2)					
1	blood, viscous	hip	*Enterobacter cloacae*	2.47	*Enterobacter cloacae*
2	blood, pus	hip	*Streptococcus agalactiae*	2.30	*Streptococcus agalactiae*
3	blood, pus	hip	*Staphylococcus aureus*	2.19	*Staphylococcus aureus*
5	blood, viscous	spine	*Staphylococcus aureus*	2.24	*Staphylococcus aureus*
6	pus	spine	*Staphylococcus aureus*	2.13	*Staphylococcus aureus*
7	blood	hip	*Streptococcus pyogenes*	2.44	*Streptococcus pyogenes*
9	blood, viscous	hip	*Enterococcus faecium*	2.00	*Enterococcus faecium*
10	blood, pus	n.r.	*Staphylococcus aureus*	2.33	*Staphylococcus aureus*
12	blood, viscous	knee	*Escherichia coli*	2.39	*Escherichia coli*
13	blood, viscous	n.r.	*Escherichia coli*	2.30	*Escherichia coli*
14	clear, viscous	knee	*Staphylococcus aureus*	2.34	*Staphylococcus aureus*
15	clear, viscous	knee	*Escherichia coli*	2.28	*Escherichia coli*
16	pus	knee	*Staphylococcus aureus*	2.37	*Staphylococcus aureus*
17	clear, viscous	knee	*Staphylococcus aureus*	2.39	*Staphylococcus aureus*
18	pus	knee	*Staphylococcus aureus*	2.37	*Staphylococcus aureus*
19	pus, viscous	n.r.	*Staphylococcus aureus*	2.29	*Staphylococcus aureus*
20	pus, viscous	hip	*Staphylococcus epidermidis*	2.16	*Staphylococcus epidermidis*
21	pus, viscous	knee	*Staphylococcus epidermidis*	2.03	*Staphylococcus epidermidis*
22	blood	knee	*Staphylococcus epidermidis*	2.23	*Staphylococcus epidermidis*
**concordant identification to species level, but only with genus level scores (≥ 1.7–1.999)**					
4	blood, pus	hip	*Staphylococcus aureus*	1.97	*Staphylococcus aureus*
8	blood, viscous	knee	*Staphylococcus epidermidis*	1.99	*Staphylococcus epidermidis*
**mixed cultures**					
11	blood, pus	n.r.	*Candida albicans / C*. *parapsilosis*	2.14	*Candida parapsilosis*
23	blood, viscous	hip	*Escherichia coli / Enterococcus faecalis*	1.57	no reliable identification

n.r. not recorded.

**Fig 1:**
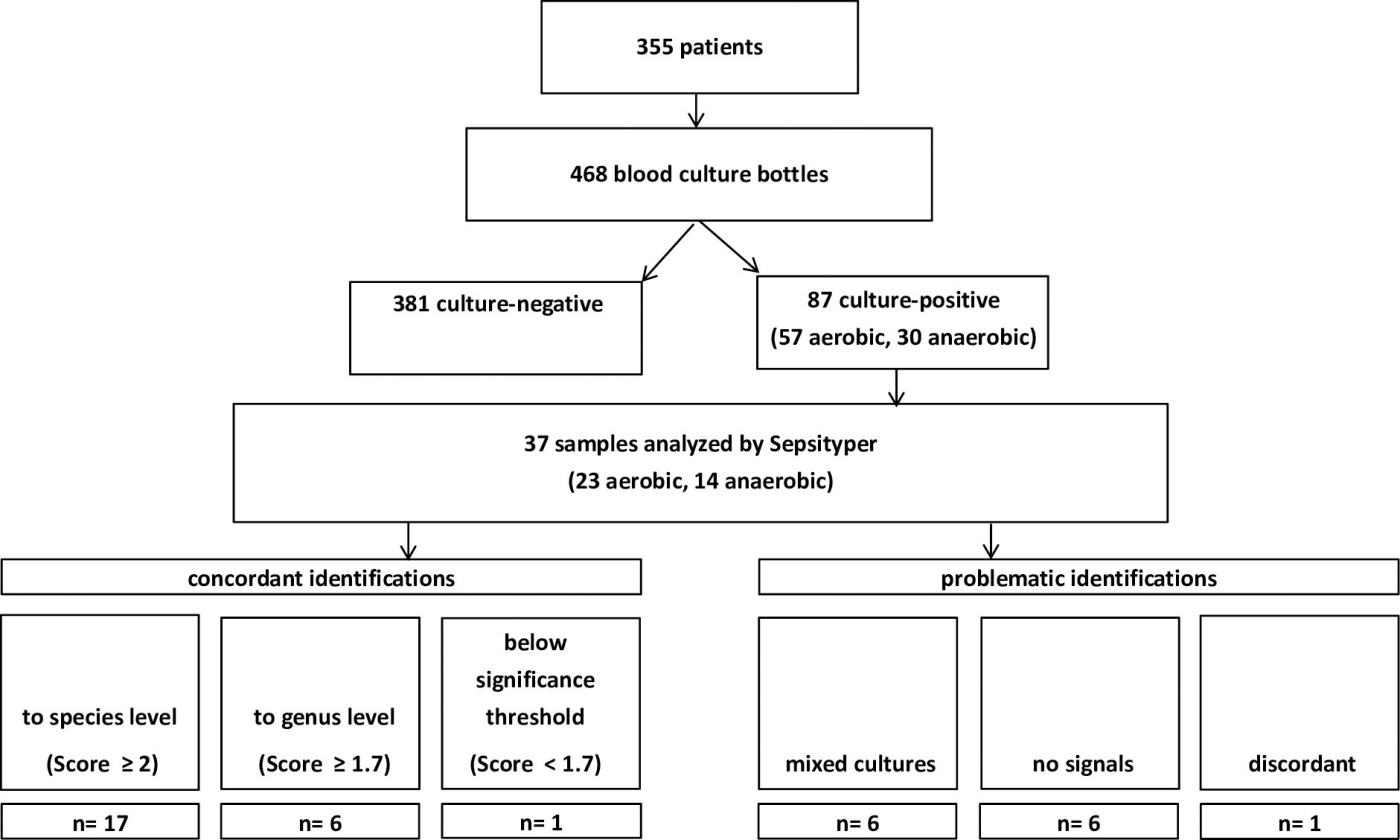
Prospective study scheme.

In order to test if this procedure also worked outside artificial laboratory conditions, we prospectively applied it on positively flagged specimen from our routine diagnostics. There, specimen had been inoculated on ward by the treating physician, and the specific condition of the inoculum was unknown (Fig 1). A total of 468 blood culture bottles inoculated with synovia from 355 clinical specimens were received during the study period, out of which 87 were flagged positive. Fifty bottles were excluded because they were not made immediately available for the study, mainly because they flagged positive during the night during the night leading to unrepresentatively high microbial yield, leaving 37 bottles to be tested by the Sepsityper workflow.

Pathogen identification concordant with results from isolates cultured on solid agar was achieved in 30/37 (81%) samples at the first try, however 7/37 (19%) again not at levels required for species-level identifications. One sample gave discordant species-level matches for the bottle (*Streptococcus mitis*) and solid agar (*Streptococcus pneumoniae*). Reliable distinction *of S*. *pneumoniae* from viridans group streptococci is important because of the different pathogenic properties of these organisms, and this has also previously been achieved by MALDI-TOF MS by others [16].

Five out of 37 samples (14%) contained mixed-species cultures of either *Staphylococcus aureus* combined with different enterobacteria, or *Pseudomonas aeruginosa* combined with *Entercoccus faecieum* (4 samples of the same patient). Mixed cultures have previously been shown to be problematic, where the ration of the organisms is unequal [17,18]. Among our samples, only in one the result indicated a mixed culture with both species, in the other four only a single species was found with high score values, however, plating of the culture indicated no more than 4-fold differences in cfu between each of the two species present. In the six remaining samples, identification was not achieved due to the complete lack of mass signals, potentially attributable to the loss of the bacterial pellet or too low cell counts in the culture (Table 2).

**Table 2:** Results of prospective Sepsityper identification.

Type of blood vial	Location/ Joint	side	bacterial species identified from direct ID by Sepsityer	MALDI-TOF score	Reference identification
**concordant species-level identification (score ≥ 2)**					
aerobic	hip	left	*Streptococcus agalactiae*	2.51	*Streptococcus agalactiae*
aerobic	knee	left	*Staphylococcus lugdunensis*	2.18	*Staphylococcus lugdunensis*
aerobic	knee	left	*Staphylococcus lugdunensis*	2.29	*Staphylococcus lugdunensis*
aerobic	hip	right	*Staphylococcus capitis*	2.19	*Staphylococcus capitis*
aerobic	elbow	left	*Staphylococcus aureus*	2.44	*Staphylococcus aureus*
aerobic	hip	left	*Streptococcus dysgalactiae*	2.10	*Streptococcus dysgalactiae*
aerobic	knee	right	*Staphylococcus capitis*	2.13	*Staphylococcus capitis*
aerobic	hip	right	*Escherichia coli*	2.41	*Escherichia coli*
aerobic	knee	right	*Streptococcus agalactiae*	2.00	*Streptococcus agalactiae*
aerobic	hip	right	*Staphylococcus aureus*	2.37	*Staphylococcus aureus*
aerobic	shoulder	right	*Streptococcus dysgalactiae*	2.01	*Streptococcus dysgalactiae*
aerobic	knee	right	*Staphylococcus epidermidis*	2.24	*Staphylococcus epidermidis*
aerobic	knee	right	*Escherichia coli*	2.56	*Escherichia coli*
anaerobic	hip	left	*Propionibacterium acnes*	2.25	*Propionibacterium acnes*
anaerobic	knee	left	*Propionibacterium acnes*	2.41	*Propionibacterium acnes*
anaerobic	knee	left	*Propionibacterium acnes*	2.37	*Propionibacterium acnes*
anaerobic	hip	right	*Enterobacter cloacae*	2.42	*Enterobacter cloacae*
anaerobic	knee	left	*Staphylococcus epidermidis*	2.11	*Staphylococcus epidermidis*
**concordant species-level identification, but only with genus level scores (≥ 1.7–1.999)**					
aerobic	hip	left	*Staphylococcus epidermidis*	1.85	*Staphylococcus epidermidis*
aerobic	knee	left	*Staphylococcus epidermidis*	1.71	*Staphylococcus epidermidis*
anaerobic	knee	left	*Staphylococcus epidermidis*	1.96	*Staphylococcus epidermidis*
anaerobic	knee	left	*Streptococcus dysgalactiae*	1.75	*Streptococcus dysgalactiae*
anaerobic	hip	left	*Propionibacterium acnes*	1.70	*Propionibacterium acnes*
aerobic	hip	left	*Streptococcus constellatus*	1.98	*Streptococcus constellatus*
**concordant species-level identification, but with scores below significance threshold (< 1.7)**					
aerobic	knee	left	*Staphylococcus epidermidis*	1.65	*Staphylococcus epidermidis*
**discordant species complex-level identifications**					
anaerobic	hip	right	*Streptococcus pneumoniae*	2.08	*Streptococcus oralis*
**Mixed cultures**					
aerobic	hip	left	*Staphylococcus aureus*	2.29	*S*. *aureus*, *E*. *cloacae*, *E*. *kobei*
aerobic	hip	right	*Pseudomonas aeruginosa*	2.23	*P*. *aeruginosa*, *E*. *faecium*
anaerobic	hip	right	*Enterococcus faecium*	2.41	*Enterococcus faecium*
aerobic	hip	right	*Enterococcus faecium*, *Pseudomonas aeruginosa*	2.17	*Enterococcus faecium*, *Pseudomonas aeruginosa*
				2.03	
anaerobic	hip	right	*Enterococcus faecium*	2.40	*Enterococcus faecium*
**no identification**					
aerobic	hip	right	no peaks found	n.a.	*Campylobacter coli*
aerobic	hip	right	no peaks found	n.a.	*Streptococcus oralis*
aerobic	hip	right	no peaks found	n.a.	*Streptococcus oralis*
aerobic	hip	right	no peaks found	n.a.	*Staphylococcus aureus*
anaerobic	shoulder	n.r.	no peaks found	n.a.	*Staphylococcus aureus*
anaerobic	knee	left	no peaks found	n.a.	*Propionibacterium acnes*

n.r., not recorded, n.a. not applicable.

## Conclusion

In summary, the Sepsityper workflow also worked well on mono-species samples derived from BacT/Alert blood culture bottles inoculated with synovia. The major contributor to failed detection was the absence of mass signals. The level of concordant identifications in both our specimen groups was comparable to those achieved by others for synovia [12], or blood cultures (summarized in [9,10]). In the case of mixed cultures, the test was not able to reliably detect the mixture, despite apparent similar cfu numbers. Host remnant material in the inoculum, such as blood or pus, had no detrimental effect on identification score values of the MALDI BioTyper system, and neither had the initial viscosity of the synovial sample.

## Materials and methods

### Sample acquisition and study design

For assessment of method feasibility, pediatric aerobic FCS-supplemented blood culture vials (BacT/ALERT PF Plus bioMérieux, Nürtingen, Germany) were inoculated with refrigerated synovial fluid that was previously found to be culture positive by our routine diagnostic procedures. Samples were selected on the basis that they had to be viscous, ideally with large proportions of blood and/or pus. Positively flagged culture vials were processed by MALDI-TOF MS as described below.

For prospective study, we used blood culture specimens, where the vials had been routinely inoculated by treating physicians with synovial fluid, transported to the microbiology lab, and cultivated in an automated microbial detection system (BacT/ALERT3D, bioMérieux) for up to 14 days. Only those samples that were made available for the study without delay were included for downstream processing by MALDI-TOF. The collection period was January 01, 2018 to March 07, 2019.

### Routine culture

Standard microbiological cultures were performed on all specimen included into the study by plating on Columbia agar supplemented with 5% sheep blood (bioMérieux) under aerobic conditions, on Chocolate agar (bioMérieux) under atmosphere enriched with 9% C0_2_ for 48h and, under anaerobic conditions on Columbia agar supplemented with 5% sheep blood (bioMérieux) for 48 h at 37°C. Reference identifications from culture plates were performed using the same MALDI Biotyper system as below, using standard procedures.

### MALDI-TOF MS analysis

Direct processing of the samples with the Sepsityper kit (Bruker Daltonics, Bremen, Germany) was carried out according to the manufacturer's instructions and as described in the literature for blood cultures [19]. In detail, 1 mL of the medium from a positive blood culture bottle was mixed with 200 μL of the lysis reagent and thoroughly mixed for 10 sec. After short incubation at room temperature, this mixture was passed over a spin column (Sigma-Aldrich, SC1000-1KT) for 2 minutes at 2000 rpm in a table top centrifuge and the filter discarded. Microbial cells were harvested from the flow through by centrifugation 1 min at 11500 x*g*. The supernatant was removed by aspiration and discarded, and the pellet washed with 1 mL saline. After further centrifugation, the supernatant was decanted and the pellet was re-suspended to 300 μL with deionized water. After addition of 900 μL 100% ethanol the cells were again harvested by centrifugation for 2 min at 11500 x*g*. The supernatant then was decanted, any residual ethanol then was removed and the pellet was dried at room temperature.

For extraction, 70% formic acid and an equal volume of 100% acetonitrile were added to the pellet and each mixed carefully. The sample was centrifuged for 2 min at maximum speed and 1 μL of the supernatant then was transferred to a sample spot on a MALDI target plate. After the sample spot had air dried it was overlaid with 1 μL matrix solution (Bruker Daltonics) and dried again. Three spots on the MALDI target plate were used for each sample. Afterwards identification of species was performed using a MALDI BioTyper SMART system using the standard settings (Bruker Daltonics, database version 2018).

### Ethical approval

This study was approved by the Ethical board on the University Medical Center Göttingen (approval number 17/11/29; version 2.0). Patients have given written consent to participate in studies.

## References

[1] Leitner L, Turk S, Heidinger M, Stockl B, Posch F, Maurer-Ertl W, et al Trends and Economic Impact of Hip and Knee Arthroplasty in Central Europe: Findings from the Austrian National Database. Sci Rep. 2018;8(1):4707 10.1038/s41598-018-23266-w 29549305 PMC5856851

[2] Metsemakers WJ, Kuehl R, Moriarty TF, Richards RG, Verhofstad MHJ, Borens O, et al Infection after fracture fixation: Current surgical and microbiological concepts. Injury. 2018;49(3):511–22. 10.1016/j.injury.2016.09.019 27639601

[3] Parvizi J, Della Valle CJ. AAOS Clinical Practice Guideline: diagnosis and treatment of periprosthetic joint infections of the hip and knee. J Am Acad Orthop Surg. 2010;18(12):771–2. 10.5435/00124635-201012000-00007 21119143

[4] Lallemand E, Coiffier G, Arvieux C, Brillet E, Guggenbuhl P, Jolivet-Gougeon A. MALDI-TOF MS performance compared to direct examination, culture, and 16S rDNA PCR for the rapid diagnosis of bone and joint infections. Eur J Clin Microbiol Infect Dis. 2016;35(5):857–66. 10.1007/s10096-016-2608-x 26942744

[5] Font-Vizcarra L, Garcia S, Martinez-Pastor JC, Sierra JM, Soriano A. Blood culture flasks for culturing synovial fluid in prosthetic joint infections. Clin Orthop Relat Res. 2010;468(8):2238–43. 10.1007/s11999-010-1254-3 20162386 PMC2895826

[6] Larsen LH, Lange J, Xu Y, Schonheyder HC. Optimizing culture methods for diagnosis of prosthetic joint infections: a summary of modifications and improvements reported since 1995. Journal of Medical Microbiology. 2012;61(Pt 3):309–16. 10.1099/jmm.0.035303-0 22222201

[7] Bogut A, Niedzwiadek J, Koziol-Montewka M, Strzelec-Nowak D, Blacha J, Mazurkiewicz T, et al Characterization of *Staphylococcus epidermidis* and *Staphyloccocus warneri* small-colony variants associated with prosthetic-joint infections. Journal of Medical Microbiology. 2014;63(Pt 2):176–85. 10.1099/jmm.0.066068-0 24257683

[8] Schafer P, Fink B, Sandow D, Margull A, Berger I, Frommelt L. Prolonged bacterial culture to identify late periprosthetic joint infection: a promising strategy. Clinical Infectious Diseases. 2008;47(11):1403–9. 10.1086/592973 18937579

[9] Scott JS, Sterling SA, To H, Seals SR, Jones AE. Diagnostic performance of matrix-assisted laser desorption ionisation time-of-flight mass spectrometry in blood bacterial infections: a systematic review and meta-analysis. Infectious diseases (London, England). 2016;48(7):530–6. 10.3109/23744235.2016.1165350 27118169

[10] Ruiz-Aragon J, Ballestero-Tellez M, Gutierrez-Gutierrez B, de Cueto M, Rodriguez-Bano J, Pascual A. Direct bacterial identification from positive blood cultures using matrix-assisted laser desorption/ionization time-of-flight (MALDI-TOF) mass spectrometry: A systematic review and meta-analysis. Enferm Infecc Microbiol Clin. 2017 10.1016/j.eimc.2017.08.012 29110928

[11] Morgenthaler NG, Kostrzewa M. Rapid identification of pathogens in positive blood culture of patients with sepsis: review and meta-analysis of the performance of the sepsityper kit. International journal of microbiology. 2015;2015:827416 10.1155/2015/827416 26000017 PMC4426779

[12] Lallemand E, Arvieux C, Coiffier G, Polard JL, Albert JD, Guggenbuhl P, et al Use of MALDI-TOF mass spectrometry after liquid enrichment (BD Bactec) for rapid diagnosis of bone and joint infections. Res Microbiol. 2017;168(2):122–9. 10.1016/j.resmic.2016.09.005 27677682

[13] Szabados F, Michels M, Kaase M, Gatermann S. The sensitivity of direct identification from positive BacT/ALERT (bioMerieux) blood culture bottles by matrix-assisted laser desorption ionization time-of-flight mass spectrometry is low. Clin Microbiol Infect. 2011;17(2):192–5. 10.1111/j.1469-0691.2010.03229.x 20370799

[14] Riederer K, Cruz K, Shemes S, Szpunar S, Fishbain JT. MALDI-TOF identification of Gram-negative bacteria directly from blood culture bottles containing charcoal: Sepsityper(R) kits versus centrifugation-filtration method. Diagnostic microbiology and infectious disease. 2015;82(2):105–8. 10.1016/j.diagmicrobio.2015.03.003 25801781

[15] Tsuchida S, Murata S, Miyabe A, Satoh M, Takiwaki M, Matsushita K, et al An improved in-house lysis-filtration protocol for bacterial identification from positive blood culture bottles with high identification rates by MALDI-TOF MS. Journal of microbiological methods. 2018;148:40–5. 10.1016/j.mimet.2018.03.014 29608928

[16] Harju I, Lange C, Kostrzewa M, Maier T, Rantakokko-Jalava K, Haanpera M. Improved Differentiation of *Streptococcus pneumoniae* and Other S. mitis Group Streptococci by MALDI Biotyper Using an Improved MALDI Biotyper Database Content and a Novel Result Interpretation Algorithm. J Clin Microbiol. 2017;55(3):914–22. 10.1128/JCM.01990-16 28053215 PMC5328460

[17] Mortelmaier C, Panda S, Robertson I, Krell M, Christodoulou M, Reichardt N, et al Identification performance of MALDI-ToF-MS upon mono- and bi-microbial cultures is cell number and culture proportion dependent. Analytical and bioanalytical chemistry. 2019;411(26):7027–38. 10.1007/s00216-019-02080-x 31486868 PMC6834929

[18] Reeve MA, Bachmann D. MALDI-TOF MS protein fingerprinting of mixed samples. Biol Methods Protoc. 2019;4(1):bpz013 10.1093/biomethods/bpz013 32395630 PMC7200911

[19] Schubert S, Weinert K, Wagner C, Gunzl B, Wieser A, Maier T, et al Novel, improved sample preparation for rapid, direct identification from positive blood cultures using matrix-assisted laser desorption/ionization time-of-flight (MALDI-TOF) mass spectrometry. J Mol Diagn. 2011;13(6):701–6. 10.1016/j.jmoldx.2011.07.004 21889611 PMC3194056

